# Pathogenic and Apathogenic Strains of Lymphocytic Choriomeningitis Virus Have Distinct Entry and Innate Immune Activation Pathways

**DOI:** 10.3390/v16040635

**Published:** 2024-04-19

**Authors:** Dylan M. Johnson, Nittaya Khakhum, Min Wang, Nikole L. Warner, Jenny D. Jokinen, Jason E. Comer, Igor S. Lukashevich

**Affiliations:** 1 Center for Predictive Medicine for Biodefense and Emerging Infectious Diseases, Louisville, KY 94202, USAigor.lukashevich@louisville.edu (I.S.L.); 2 Department of Microbiology and Immunology, University of Louisville Health Sciences Center, Louisville, KY 94202, USA; 3 Galveston National Laboratory, Department of Microbiology & Immunology, University of Texas Medical Branch, Galveston, TX 77550, USA; nikhakum@utmb.edu (N.K.); jason.comer@lsuhs.edu (J.E.C.); 4 Sandia National Laboratories, Department of Biotechnology & Bioengineering, Livermore, CA 94550, USA; 5 Department of Pharmacology and Toxicology, University of Louisville Health Sciences Center, Louisville, KY 94202, USA; min.wang@louisville.edu

**Keywords:** lymphocytic choriomeningitis virus, toll-like receptors, intracellular trafficking

## Abstract

Lymphocytic choriomeningitis virus (LCMV) and Lassa virus (LASV) share many genetic and biological features including subtle differences between pathogenic and apathogenic strains. Despite remarkable genetic similarity, the viscerotropic WE strain of LCMV causes a fatal LASV fever-like hepatitis in non-human primates (NHPs) while the mouse-adapted Armstrong (ARM) strain of LCMV is deeply attenuated in NHPs and can vaccinate against LCMV-WE challenge. Here, we demonstrate that internalization of WE is more sensitive to the depletion of membrane cholesterol than ARM infection while ARM infection is more reliant on endosomal acidification. LCMV-ARM induces robust NF-κB and interferon response factor (IRF) activation while LCMV-WE seems to avoid early innate sensing and failed to induce strong NF-κB and IRF responses in dual-reporter monocyte and epithelial cells. Toll-like receptor 2 (TLR-2) signaling appears to play a critical role in NF-κB activation and the silencing of TLR-2 shuts down IL-6 production in ARM but not in WE-infected cells. Pathogenic LCMV-WE infection is poorly recognized in early endosomes and failed to induce TLR-2/Mal-dependent pro-inflammatory cytokines. Following infection, Interleukin-1 receptor-associated kinase 1 (IRAK-1) expression is diminished in LCMV-ARM- but not LCMV-WE-infected cells, which indicates it is likely involved in the LCMV-ARM NF-κB activation. By confocal microscopy, ARM and WE strains have similar intracellular trafficking although LCMV-ARM infection appears to coincide with greater co-localization of early endosome marker EEA1 with TLR-2. Both strains co-localize with Rab-7, a late endosome marker, but the interaction with LCMV-WE seems to be more prolonged. These findings suggest that LCMV-ARM’s intracellular trafficking pathway may facilitate interaction with innate immune sensors, which promotes the induction of effective innate and adaptive immune responses.

## 1. Introduction

Lymphocytic choriomeningitis virus (LCMV), the prototypical member of the *Arenaviridae* family, is widely spread across continents and typically causes asymptomatic or mild infections, although these can progress to aseptic meningitis or meningoencephalitis [1,2,3]. During the third trimester of pregnancy, LCMV can cause infection with disastrous consequences for the fetus [1,4]. LCMV and Lassa virus (LASV), which are closely related *Mammarenaviruses*, share major biological features including interaction with a common cellular receptor, α-dystroglycan (α-DG) [5], persistence in natural rodent hosts, and pathogenicity in experimentally infected guinea pigs and non-human primates (NHPs) [6,7]. Both virus species, *Lymphocytic choriomeningitis mammarenavirus* and *Lassa mammarenavirus*, comprise a collection of highly diverse virus isolates from both rodents and humans, which can be phylogenetically placed into four and seven lineages, respectively [8,9,10,11,12,13,14]. In endemic areas of West Africa, LASV causes infection with broad clinical manifestations, from sub-clinical or mild flu-like disease to severe Lassa fever (LF) with fatal outcomes [15,16]. Furthermore, LCMV can cause fatal LF-like infection in immunocompromised tissue transplant recipients that receive LCMV-infected donor organs [17,18].

LCMV strains are endemic in the house mouse, Mus musculus. In mice, intravenous inoculation of LCMV-WE, but not LCMV-ARM, induced transient hepatitis [19]. LCMV-induced hepatitis was proposed as a model of T cell-mediated liver pathology [20]. A later study documented that cycle-arrested hepatocyte proliferation and apoptosis induced by LCMV-WE, but not LCMV-ARM, also contributed to experimental LCMV hepatitis [21]. Notably, converse to the pathogenicity of LCMV in guinea pigs and NHPs, disease outcome in the natural murine host is a complex story that is dependent on viral strain, route of infection, sex, and age at infection [22,23,24,25,26]. The LCMV Armstrong strain (LCMV-ARM) is a commonly studied, mouse-adapted, “neurotropic” isolate that does not induce clinical disease in guinea pigs nor NHPs. Conversely, the “viscerotropic” WE54 strain of LCMV (LCMV-WE) is highly pathogenic for these animals [6,24]. In Rhesus macaques, LCMV-WE induces fatal disease resembling LF-like viral hepatitis [27,28]. In contrast, LCMV-ARM (strain 53b) induces sub-clinical infection resulting in effective cell-mediated immunity, and protection against subsequent challenge with LCMV-WE [29]. Similarly, various LASV isolates demonstrate highly variable pathogenicity in humans, NHP models, and rodent models with pathogenicity ranging from fulminant hemorrhagic fever disease, with or without pulmonary and neurological involvement, to asymptomatic transient infection [30,31].

It was documented that early events of mammalian arenavirus replication are sensitive to lysosomotropic compounds, suggesting that pH-dependent endocytosis plays a role in the entry of these viruses into susceptible cells [32]. However, in the case of LCMV and LASV, this endocytosis is unusual because viral entry following binding to α-DG occurs in a host clathrin- and dynamin-independent manner [33,34,35]. Recently, micropinocytosis has been proposed as an alternative minor entry pathway of these viruses [36,37]. It has been observed that lysosome-associated membrane protein-1 (LAMP-1) triggers low-pH fusion in LASV-infected cells but not in LCMV-infected cells [38,39], which provides additional evidence that the mechanisms of LCMV and LASV entry are complex and need further elucidation. Indeed, it has been documented that additional cellular receptors and/or attachment factors such as DC-SIGN, LSECtin, heparin sulfate, TAM (Tyro3/Axl/Mer), and TIM-1 are involved in virus entry in an α-DG-independent manner [40,41,42,43,44,45]. Interestingly, in animal models of arenavirus-induced hepatitis, LCMV-WE54 infection of C57Bl/6 mice and LASV-Josiah infection of marmosets, infection was associated with up-regulation of the Axl-1 gene in hepatocytes of infected animals [21,46]. Expression of TAM and TIM-1 receptors on cell-derived viral surfaces mimics “apoptotic bodies” and promotes binding and internalization via “apoptotic mimicry” mechanisms. It has been documented that activation of TAM receptors by ligand binding has down-regulated TLR-mediated innate immune responses and IFN type I signaling, which provides a beneficial environment for viral replication [47,48,49].

After pH-dependent fusion in late endosomes, mammalian arenaviruses are sensed by endosomal pattern recognition receptors (PRRs), TLR-7 and TLR-9, and by a cytosolic PRR, retinoic acid-inducible gene I (RIG-I), via recognition of viral RNP/RNA released into the cytoplasm of plasmacytoid (pDCs). LCMV nucleoprotein (NP) and RING-finger matrix protein (Z) both play a role in immune evasion through NP-mediated degradation of dsRNA preventing activation of RIG-I; NP-mediated inhibition of the NF-κB-activating kinase IKKε; and Z-mediated interference of RIG-I binding to Mitochondrial AntiViral Signaling (MAVS) [50,51,52,53]. However, these interactions are down-stream of TLR-mediated innate immune responses and are insufficient to fully describe the differences in the complex kinetics of interferon activation observed in pathogenic and apathogenic arenaviruses [54,55]. In addition, TLR-2, widely expressed on conventional DCs (cDCs) and macrophages, is also involved in virus recognition and the induction of innate and adaptive responses. We previously documented that non-pathogenic Mopeia virus (MOPV), a close genetic relative of LASV, and LCMV-ARM53b both induced robust TLR2/Mal(MyD88 adaptor-like)-dependent and NF-κB-mediated pro-inflammatory cytokines in human epithelial cells, monocytes, and in murine macrophages [56]. In contrast, the replication of pathogenic LASV (Josiah) and LCMV-WE54 have down-regulated innate pro-inflammatory responses in vitro and in vivo [27,28,56,57,58].

Here, to further elucidate mechanisms driving host-response differences between pathogenic and non-pathogenic mammalian arenaviruses, we used different experimental tools targeting virus–cell interactions and intracellular trafficking of LCMV-ARM, which is apathogenic in NHP models, and LCMV-WE, causing fatal LF-like infection in NHPs. We found that chemical antivirals with known mechanisms of action targeting the cell surface, microtubules, and cell membrane fusion in late endosomes, expressed LCMV strain-specific pattern of antiviral activities. We have also found evidence that the NP of LCMV-ARM seemed to coordinate a co-localization of the early endosome markers, EEA1, with TLR-2; while in the late endosomes, both LCMV strains, ARM and WE, similarly co-localized with Rab7. In addition, LCMV-ARM NF-κB activation is dependent on TLR-2 and IRAK-1 and seems to contribute to the down-regulation of NF-κB-mediated signaling for LCMV-WE, which we previously documented [56].

## 2. Materials and Methods

### 2.1. Cells and Viruses

Vero E6 C1008 (ATCC CRL-1586) and RAW264.7 (ATCC TIB-71™) cells were purchased from American Type Culture Collection (Gaithersburg, MD, USA). THP1-Dual and A549-Dual cells were purchased from InvivoGen (San Diego, CA, USA). Viral stocks were produced by infecting Vero E6 cells with LCMV-Armstrong (ARM, strain 53b) or LCMV-WE (strain 54) at a low (0.01–0.001) MOI, resulting in stock titers of 1–5 × 10^7^ PFU/mL [59]. Infectious virus titration was performed in Vero E6 cells by standard plaque assay with 0.5% Avicel (FMC BioPolymer, Philadelphia, PA, USA) as previously described [60] with a limit of detection of approximately 80 PFU/mL. All work with infectious LCMV-WE was performed in BSL3 containment at either the NIH Regional Biosafety Laboratory run by the Center for Predictive Medicine on the University of Louisville campus or at the Galveston National Laboratory on the University of Texas Medical Branch campus according to protocols approved by their respective Institutional Biosafety Committees.

### 2.2. Treatment with Chemical Inhibitors

Treatment with Mβ-CD, nocodazole, and bafilomycin was performed as previously described [33,35,39,52,61,62,63,64] using Vero E6 cells seeded in 12-well tissue culture plates. Briefly, cells were pre-treated in the presence or absence (control cells) of 5 mM Mβ-CD (Sigma-Aldrich, St. Louis, MO, USA) for 1 h at 37 °C before infection with an MOI of 0.3 PFU/cell and incubated for 24 h in the presence of Mβ-CD. Cells pre-treated with 10 μM nocodazole for 1 h at 37 °C were washed 2 times with cold PBS, and infected with an MOI of 0.3 PFU/cell for 1 h before incubation in the presence of fresh nocodazole for 24 h at 37 °C. Cells were pre-treated with bafilomycin A1 (Sigma-Aldrich, St. Louise, MO, USA) before infection at an MOI of 1.0 PFU/cell at 4 °C for 1 h followed by 24 h at 37 °C in the presence of bafilomycin. After treatment, supernatants were collected, and the infectious viral titer was determined by plaque assay. Percent replication after inhibition was calculated as the percent reduction in viral titer in supernatants from treated cells compared to untreated cells. The remaining cells from the inhibition assays were lysed in RNA-STAT-60 (Tel-Test Inc., Friendswood, TX, USA) and RNA was isolated according to the manufacturer’s instructions. qRT-PCR was used to quantitate viral RNA in treated and untreated cells using LCMV strain-specific primers/probe as previously described [60].

### 2.3. IRF and NF-κB Activation in Dual-Reporter Cell Lines

THP1-Dual or A549-Dual cells were seeded in 96-well plates and infected with 1 PFU/mL MOI of either LCMV-Arm or LCMV-WE, or treated with 100 ng/mL of the RIG-I-Like-Receptor (RLR) agonist Poly(dA:dT) pre-complexed with LyoVec transfection reagent (tlrl-patc, InvivoGen, San Diego, CA, USA) as a positive control for IRF reporter activation, or 1 ng/mL for THP1-Dual or 300 ng/mL for A549-Dual of the TLR2 agonist PAM3CSK4 as a positive control for NFκB activation. Forty-eight hours after treatment, supernatants were harvested and NFκB and IRF reporter activation were quantified on a Cytation 7 absorbance reader and luminometer (Agilent, Santa Clara, CA, USA) using Quanti-Blue and Quanti-Luc reporter systems according to the manufacturer’s directions (InvivoGen, San Diego, CA, USA). For NF-κB activation, the levels of SEAP were determined by reading the optical density at 655 nm. For IRF activation, the levels of Lucia luciferase were determined by measuring the relative light. Fold changes compared to uninfected cells are reported to standardize between experiments.

### 2.4. TLR-2 Silencing in LCMV-Infected Cells

RAW264.7 cells were pre-treated with TLR2 *Silencer^®^* select pre-designed siRNA (Ambion, P/N 4390771, Waltham, MA, USA). Transfection was performed with Lipofectamine (Lipofectamine RNAi Max Reagent, Invitrogen, Waltham, MA, USA) for 48 h. Control cells were transfected with *Silencer^®^* Negative Control siRNA (Ambion). Transfection of mock-infected cells with TLR2 siRNA resulted in suppression of TLR-2 mRNA and TLR-2 expression on cell surface as assessed by staining transfected cells with anti-TLR-2 antibody and flow cytometry. Transfected cells were infected with LCMV stains (ARM and WE) at an MOI of 2–5 and incubated in a CO_2_ incubator for 24 h. The mRNA levels of IL-6 in infected cells were determined by real-time PCR using commercial primers/probe as previously described [1]. Expression of IL-6 at protein level in culture medium of infected cells was analyzed by ELISA kit (eBioscience™, P/N 88-7064).

### 2.5. Immunofluorescence Co-Staining, Confocal Microscopy

RAW264.7 cells were grown in 12-well plates containing round coverslip (Thermo Fisher Scientific, Waltham, MA, USA) and infected with LCMV strains at an MOI of 5. After virus internalization, intracellular trafficking of LCMV infection was monitored in co-staining experiments using monoclonal antibody against conservative NP epitope (M104, Abcam, 1:1000 dilution, Cambridge, UK), EEA1 (monoclonal F.43.1,Thermo scientific, 1:100), Rab5 (polyclonal, ab13253, Abcam, 1:80), Rab7 (rabbit monoclonal EPR7589, Abcam, 1:100), LAMP-1 (polyclone C-20, Santa Cruz, 1:100), TLR-2 (monoclonal CD282, eBioscience, 1:100), and IRAK-1 (D51G7, Cell Signaling, 1:250). Infected cells were incubated in a CO_2_ incubator for 30 to 120 min as indicated, fixed with 4% PFA, and permeabilized with 0.1% Triton X-100. After blocking of non-specific binding with 1% BSA, cells were co-stained with primary antibodies for 1 h at 25 °C before treatment with secondary IgG-TR or IgG-FITC antibodies (anti-rabbit, 1:1000 dilution for 1 h at 25 °C). Coverslips were mounted with DAPI Flu–G (Southern Biotech, Birmingham, AL, USA). Confocal images were acquired and analyzed on a Zeiss LSM 880 with a 60× oil-immersion objective at the UTMB Optical Microscopy core.

### 2.6. Western Blot Analysis of IRAK-1 Expression

RAW267.7 cells were grown in T25 flasks and infected with LCMV virus strains at an MOI of 0.1. At different timepoints post-infection, protein extracts were prepared and subjected to SDS-PAGE analysis as previously described [65]. In brief, protein samples were combined with 4× Laemmli sample buffer and loaded onto SDS-polyacrylamide gels of 10% and 15% (*w*/*v*) acrylamide followed by electrophoresis and Western blotting onto PVDF membranes. Primary antibodies against IRAK-1 (D51G7, Cell Signaling) and GAPDH (sc-25778, Santa Cruz Biotechnology) were used at dilutions of 1:1000 and 1:2000, respectively. Bands were visualized using horseradish peroxidase-coupled secondary antibodies, ECL kit (Pierce, Rockford, IL, USA), and Hyperfilm (GE Healthcare, Piscataway, NJ, USA). Densitometry analysis was performed using UN-SCAN-IT gel software version 7 (Silk Scientific Inc., Orem, UT, USA).

### 2.7. Statistical Analyses

Statistical significance was analyzed as reported in figure legends with GraphPad Prism (GraphPad Software version 10, San Diego, CA, USA).

## 3. Results

### 3.1. Probing LCMV-ARM versus LCMV-WE Entry with Chemical Inhibitors

Chemical antivirals with known mechanisms of action are a useful tool to study virus–cell interaction and cell factors’ involvement in virus replication [66]. In this study, representatives of three groups of drugs targeting the cell surface, microtubules, and cell membrane fusion in late endosomes were used to assess the effect of LCMV strain-specificity on antiviral drug activity.

**Figure 1:**
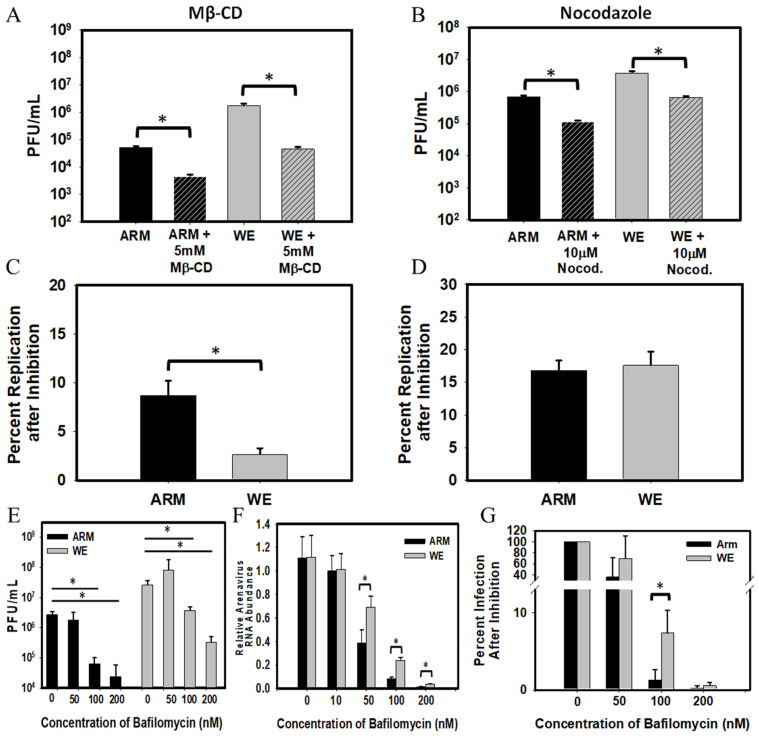
LCMV strain-specific replication sensitivity to entry and trafficking inhibitors. Vero E6 cells were treated for 1 h prior to infection with 5mM Mβ-CD (**A**,**C**) or with 10μM nocodazole (**B**,**D**), then infected with LCMV with a 0.3 MOI. Supernatants were collected 24 h after infection and virus production was determined by plaque assay (**A**,**B**). Values are shown as the mean of three biological replicates with the error bar representing the standard deviation. Percent replication after inhibition is the percentage of replication in wells containing each inhibitor normalized to corresponding control wells without inhibitor (**C**,**D**). Vero E6 cells were pre-treated with bafilomycin A1 and infected with LCMV strains ARM and WE at an MOI of 1 at 4 °C for 1 h followed by incubation for 24 h at 37 °C in the presence of bafilomycin. After treatment, supernatants were collected, and (**E**) the infectious viral titer was determined by plaque assay or (**F**) viral RNA relative was determined by qRT-PCR for each condition compared to infection with the absence of bafilomycin A1 treatment. (**G**) The percentage of plaque-forming particles detected in bafilomycin-treated cells normalized to those in bafilomycin A1 untreated cells infected with the same LCMV strain. Values are the mean of three biological replicates with the error bars representing the standard deviation. A Student’s *t*-test was used to analyze differences (* *p* ≤ 0.05).

It has been documented that the entry of LCMV (ARM-derived Clone 13) was dependent upon membrane cholesterol [62]. Using the same protocol, pre-treatment with methyl-β-cyclodextrin (Mβ-CD) to deplete cholesterol from the cell surface resulted in reduced viral titers in cells infected with both LCMV-ARM and -WE (Figure 1A). However, WE infection was significantly more sensitive to membrane cholesterol depletion than ARM infection (Figure 1C).

It was documented that the early steps of LCMV-ARM infection were actin-independent but still required intact microtubule networks [35]. In line with this observation, replication of both strains of LCMV was sensitive to nocodazole, a drug responsible for disrupting microtubular structures, and we did not find strain-specific differences in sensitivity to the antiviral activity of nocodazole (Figure 1B,D).

Fusion of arenaviruses with cell membranes is the last step of intracellular trafficking of the virus-containing vesicles. A low-pH-mediated environment is required for fusion in late endosomes/lysosomes and can be affected by drugs that increase the pH in these sub-cellular compartments either directly (e.g., ammonium chloride) or indirectly by inhibiting lysosomal proton pump V-ATPases with bafilomycin A1 [32,61]. As expected, bafilomycin A1 treatment inhibited replication of both strains of LCMV in a dose-dependent manner (Figure 1E,F). However, this inhibition was significantly more impactful to LCMV-ARM infection (Figure 1G), indicating greater sensitivity of LCMV-ARM infection to pH increase in late endosomes compared to LCMV-WE.

### 3.2. Innate Immune Activation in Cells Infected with LCMV-ARM versus LCMV-WE

To assess the ability of LCMV infection to induce innate immunity, we used two cell lines, human airway epithelial cells (A549) and human monocytes (THP1). Previous studies documented that LCMV-, ARM and WE, replicated in these cells with the same kinetics [56,60]. These cell lines were stably transfected with reporter genes, allowing the simultaneous study of the IRF pathway, by monitoring the activity of an inducible luciferase under the control of an ISG54 minimal promoter in conjunction with five IFN-stimulated response elements, and the NF-κB pathway by monitoring SEAP, inducible secreted alkaline phosphate, under the control of the IFN-β minimal promoter fused to five NF-κB binding sites. Cells were infected with LCMV-ARM and LCMV-WE at the same MOI and luciferase and SEAP were measured at 48 h after infection to assess the activation of IRF or NF-κB pathways.

**Figure 2:**
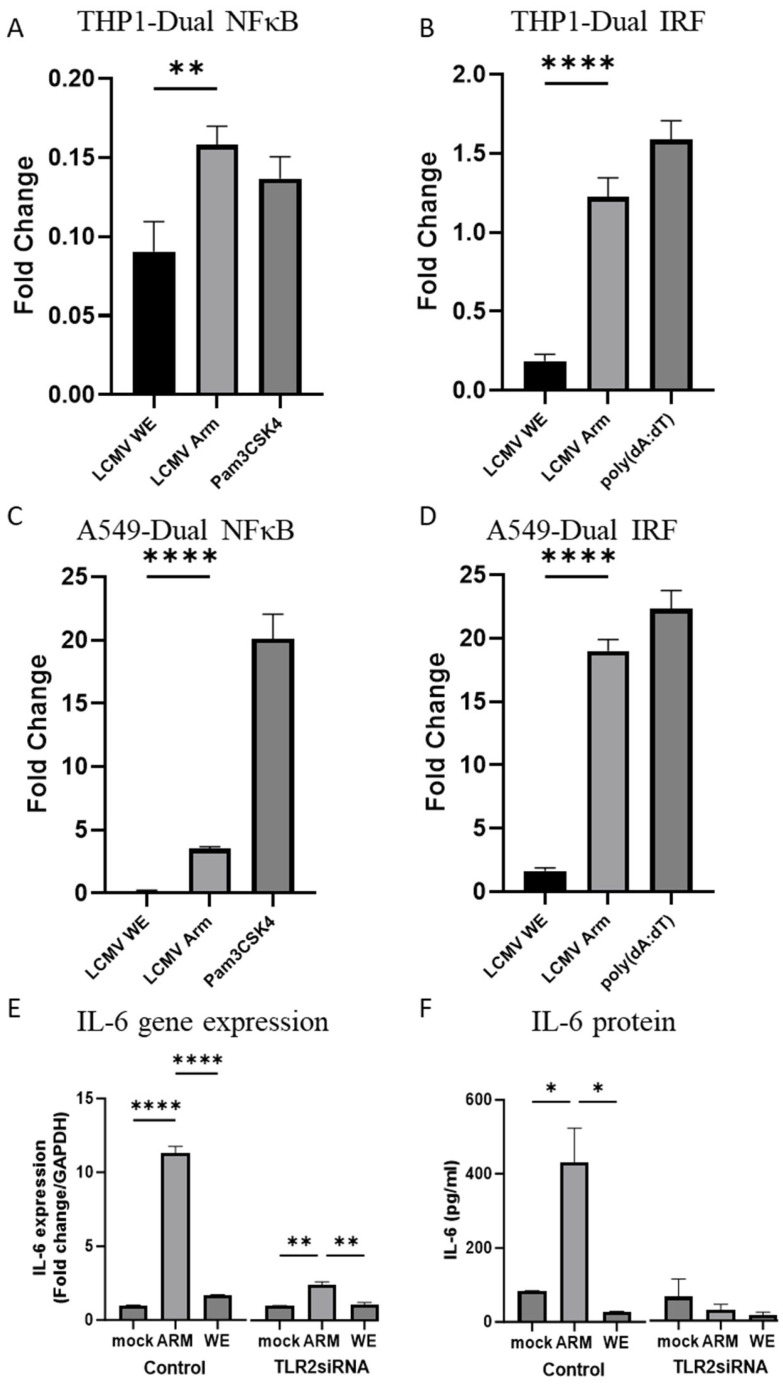
LCMV strain-specific induction of innate immune responses. THP1-Dual cells (**A**,**B**) or A549-Dual cells (**C**,**D**) were infected with either LCMV-Arm or LCMV-WE at an MOI of 1, stimulated with TLR2 agonist PAM3CSK4 as a positive control for NFκB activation, stimulated with poly(dA:dT)/LyoVec as a positive control for IRF activation, or left uninfected. After 48 h incubation, NF-κB (**A**,**C**) and IRF (**B**,**D**) activation were assessed compared to uninfected controls. Values are reported as log_2_ fold change. Each bar represents two independent experiments with six or eight biological replicates each and error bars representing the standard error of the means. Student’s *t*-tests were used to compare LCMV-Arm to LCMV-WE (** *p* < 0.01, ****, *p* < 0.0001). IL-6 mRNA expression (**E**) in infected (control) and TLR2 siRNA transfected cells with LCMV-ARM, LCMV-WE, or mock infection determined by qRT-PCR. Protein levels of IL-6 (**F**) in culture medium of cells was measured with a mouse IL-6 ELISA kit. Data (triplicate/group) represented as mean with standard deviation error bars, a one-way ANOVA with Tukey’s post-hoc analysis was used to compare the means within each experimental condition (* *p* < 0.05, ** *p* < 0.01, **** *p* < 0.0001.).

As seen in Figure 2A–D, LCMV-ARM more efficiently activated NF-κB signaling in both epithelial- and monocyte-derived cell lines. Studies in TLR2 and Mal knock-out mice supported in vitro findings that documented the crucial role of TLR2/Mal-dependent signaling in the increased NF-κB activation caused by LCMV-ARM infection compared to LCMV-WE [56]. To confirm this phenotype, TLR-2 siRNA was transfected to silence TLR-2 gene expression and subsequently, cells were infected with both LCMV strains. In line with the previous findings [56], TLR-2 silencing dramatically down-regulated IL-6 gene expression and secretion of IL-6 protein in cells infected with LCMV-ARM. As expected, LCMV-WE infection did not induce IL-6 and the effect of TLR-2 silencing in WE-infected cells was minimal if any (Figure 2E,F).

LCMV strain-specific effects were prominent on IFR activation pathways. In monocyte-derived cells, LCMV-ARM infection strongly activated the reporter gene at a scale comparable to the effects of a classical IRF inducer, poly (dA:dT). In contrast, LCMV-WE infection had a negligible effect on reporter activity. Together, LCMV-ARM infection of dual-reporter cell systems resulted in the stronger activation of NF-κB and IFN pathways compared to LCMV-WE infection.

### 3.3. Co-Staining Experiments with Markers of LCMV Infection, TLR-2, and Early Endosomes

**Figure 3:**
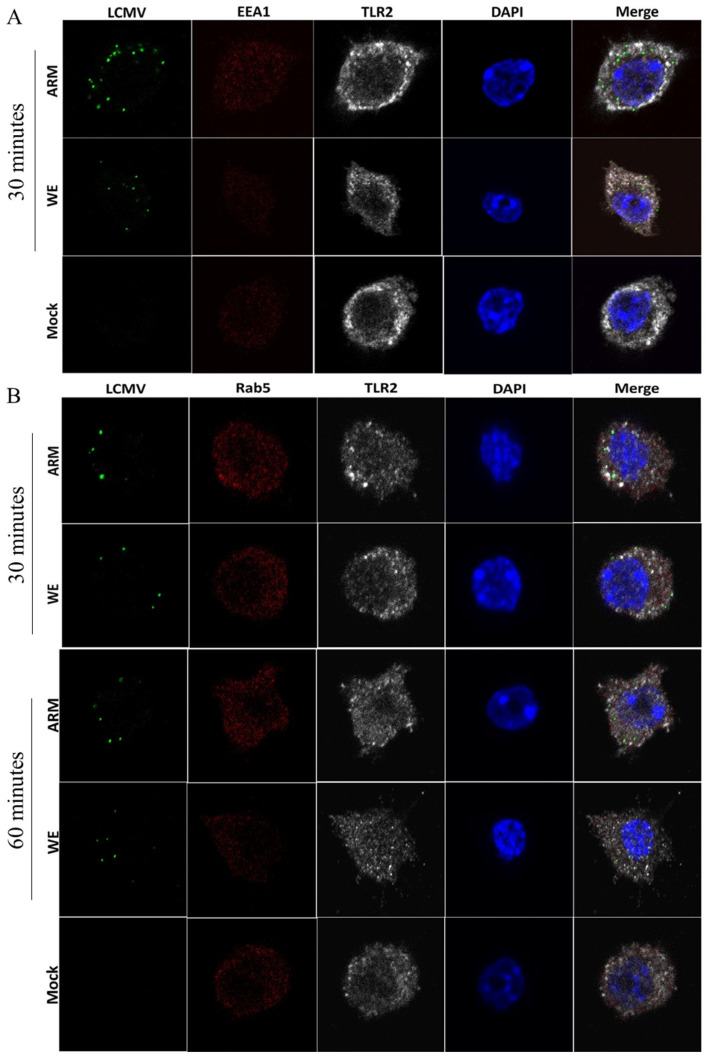
Co-staining of LCMV NP with markers of early endosomes and TLR-2. RAW 264.7 cells were infected with LCMV (ARM or WE) at an MOI of 5 and incubated for 30 or 60 min as indicated before being fixed, permeabilized, and stained with antibody against a conserved LCMV NP epitope (monoclonal M104, green), markers of early endosomes, (**A**) EEA1 (monoclonal F.43.1, red) or (**B**) Rab5 (polyclonal ab13253, red), and TLR2 (monoclonal CD282, grey). Nuclei of cells were stained with DAPI (blue).

After internalization, LCMV and LASV virions are sorted into endocytic vesicles and delivered to endosomal/lysosomal compartments via endocytosis and micropinocytosis [37,45]. To assess the interaction between virus-loaded vesicles and traffic through endosomal TLR-containing compartments, mock- and LCMV-infected (RAW264.7) macrophages were stained with monoclonal antibody M104 targeting a conserved LCMV NP epitope along with antibodies targeting Rab5 and EEA1 markers of endosomal compartments, and the co-staining pattern was assessed by confocal microscopy. Additionally, TLR-2, which is associated with early endosomes and involved in arenavirus sensation, was also included in co-staining experiments (Figure 3).

**Figure 4:**
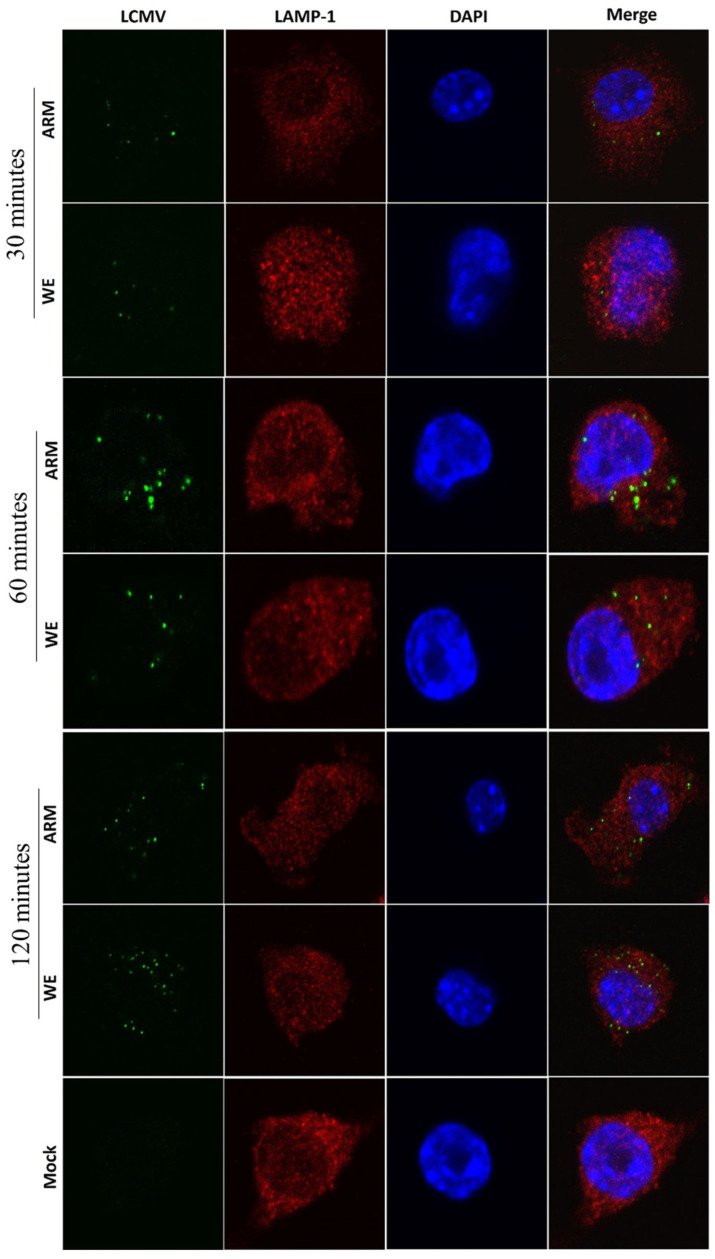
Co-staining of LCMV-infected cells for NP and LAMP-1 antibodies. RAW 264.7 cells were infected with LCMV (ARM or WE) at an MOI of 5 and incubated for 30, 60, or 120 min as indicated before being fixed, permeabilized, and stained with antibody against a conserved LCMV NP epitope (green), LAMP-1 (red), and DAPI (blue). No co-localization signals were detected at indicated timepoints after infection.

**Figure 5:**
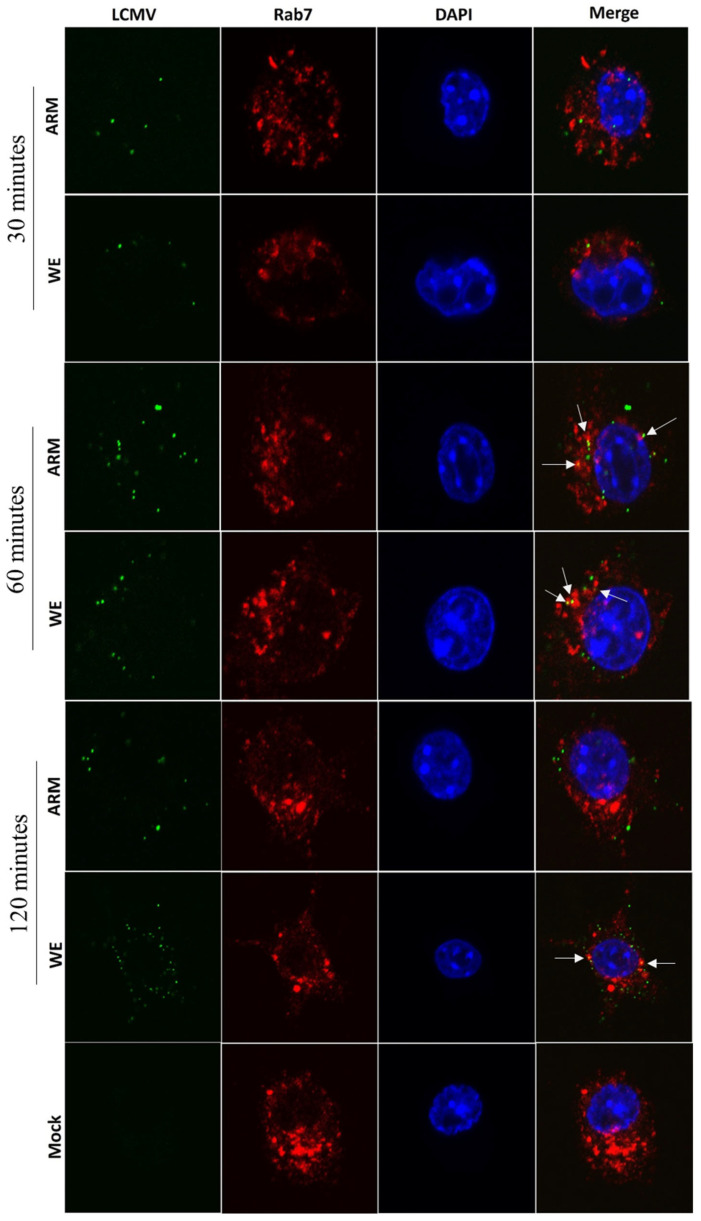
Co-localization of LCMV antigen with Rab7. RAW 264.7 cells were infected with LCMV (ARM or WE) at an MOI of 5 and incubated for 30, 60, or 120 min as indicated before being fixed, permeabilized, and stained with antibody against a conserved LCMV NP epitope (green), Rab7 (red), and DAPI (blue). Co-localization signals, yellow spots, are indicated with white arrows. For LCMV-ARM infection, co-localization signals were transiently detected 60 min after infection. For LCMV-WE, in addition to 60 min exposure, yellow spots were seen and at the later timepoint.

**Figure 6:**
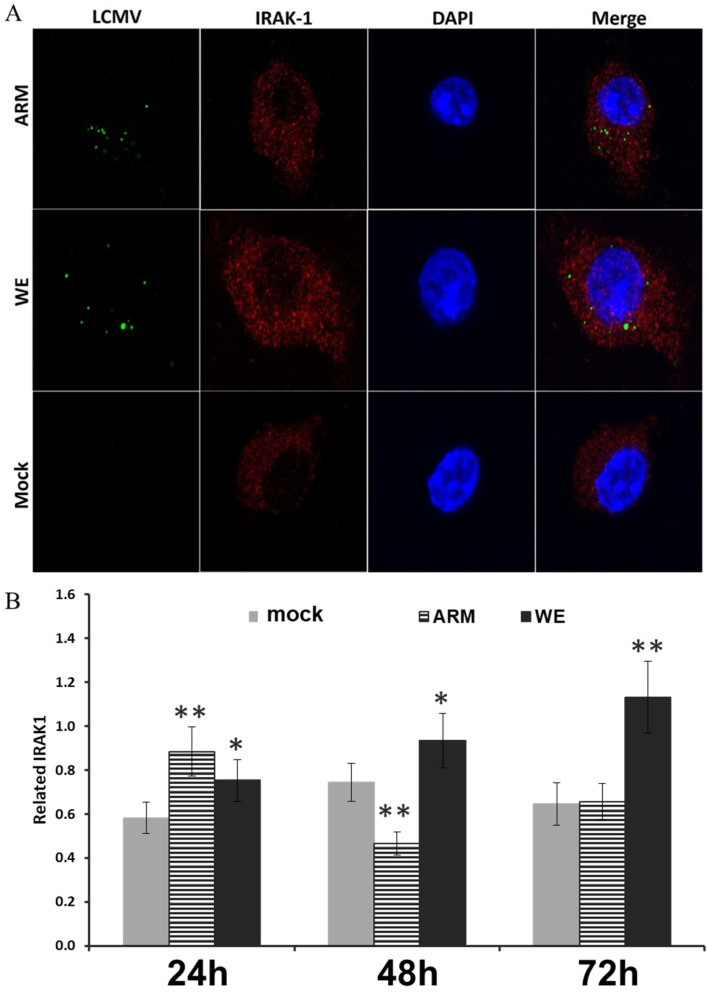
IRAK-1 expression in LCMV-infected cells. (**A**) RAW 264.7 were infected with LCMV (ARM or WE) at an MOI of 5 and incubated for 60 min before being fixed, permeabilized, and stained with antibody against a conserved LCMV NP epitope (green), IRAK-1 (red), and DAPI (blue). (**B**) IRAK-1 protein in control and infected cells was identified as an 80 kDa band. Density of IRAK-1 bands from three independent experiments was normalized to GAPDH and quantified with UN-SCAN-IT gel software version 7. Results are presented with standard error of the mean error bars and significance indicators based on Student’s *t*-test comparison to mock infection (* *p* < 0.05, ** *p* < 0.01).

M104 equally stained ARM- and WE-infected cells and produced punctate staining indicative of replication and transcription complexes (RTCs) similar to previous observation [67] as early as 30 min following infection (Figure 3, Figure 4, Figure 5 and Figure 6). In co-staining experiments with markers of early endosomes and TLR-2, no clear differences in LCMV NP staining patterns and no clear co-staining of viral NP with TLR-2, EEA1, or Rab5 were observed for either ARM- or WE-infected cells (Figure 3). There were also no noticeable differences in Rab5 and TLR-2 co-localization patterns following infection (Figure 3B). Nevertheless, there seemed to be a small but noticeable increase in the co-staining of EEA1 with TLR-2 in LCMV-ARM-infected cells compared to LCMV-WE or mock infection (Figure 3A).

### 3.4. LCMV Interaction with Markers of Late Endosomes/Exosomes

LASV and LCMV glycoproteins fusion occurs at a very low pH (3.0–4.5), suggesting involvement of the lysosomal compartment [61]. A unique feature of LASV is the usage of the second receptor, LAMP-1 [38,39], to promote fusion in the less acidic compartments [68]. Notably, a low pH requirement for LCMV-ARM-mediated fusion was not dependent on LAMP-1 expression [68]. In line with these findings, co-staining for LCMV NP and LAMP-1 demonstrated similar staining patterns for ARM- and WE-infected cells at 30, 60, and 120 min post-infection (Figure 4) with no evidence of co-staining interaction. These experiments provide additional evidence that in contrast to LASV infection, LAMP-1 is not required for LCMV-induced fusion.

For several viruses, Rab7-related trafficking has been associated with cytosolic entry and late steps of virus replication, assembly, and release [69]. The low pH environment of late endosomes promotes the fusion of arenaviruses with the cell membrane and the release of their RNA genome into the cytoplasm. Co-staining of LCMV and Rab7 in infected cells provided evidence of co-localization at 60 min post-infection. As seen in Figure 5 (white arrows), clear evidence of LASV NP and Rab7 co-staining was documented for LCMV-ARM infection. For LCMV-WE, a weak signal co-staining was also seen at a 60 min timepoint and was increased at a 120 min timepoint. The ARM-infected cells did not have noticeable co-staining at this late timepoint.

### 3.5. Interaction with IRAK-1, Mediator of TLR-Induced Signaling, in LCMV-Infected Cells

TLR-2 signaling is dependent on the recruitment of several key adaptor molecules. Upon ligand recognition, MyD88 (and/or Mal) recruits and activates IL-1-associated kinases (IRAK), such as IRAK-1, triggering a down-stream activation cascade, leading to NF-kB translocation and transcription initiation [70]. As shown above (Figure 2), in cells transfected with a NF-κB–luciferase reporter, infection with LCMV-ARM resulted in the stronger induction of NF-κB than in cells infected with LCMV-WE.

To address IRAK-1 involvement in down-stream TLR-2 signaling, LCMV-infected macrophages were co-stained with LCMV NP and IRAK-1 antibodies 60 min post-infection. As seen in Figure 6A, similar staining signals were detected for both LCMV-ARM- and LCMV-WE-infected cells.

In response to stimulation, IRAK-1 is subjected to ubiquitination, degradation, and IRAK-1 protein levels remain suppressed up to 8 h after stimulation [71]. While there was no difference between expressions of IRAK-1 protein (in Western blot) at 24 h after infection in ARM- and WE-infected cells, the IRAK-1 expression levels were decreased at later timepoints in ARM-infected cells in comparison with mock- and WE-infected cells (Figure 6B).

## 4. Discussion

In this study, we hypothesized that while the general mechanism of LCMV entry is common for both ARM and WE strains of LCMV, the intracellular trafficking and interactions with markers of endosomal compartments (e.g., EEA1, Rab5, Rab7, LAMP-1) and PPRs (e.g., TLR-2) are strain-specific events that contribute to different patterns of innate immune responses and pathogenicity of LCMV-induced disease in experimental animals. In humans and NHPs, LCMV-WE induces fatal hepatitis, but LCMV-ARM is deeply attenuated and protects NHPs against fatal WE [27,57,72]. Similarly, guinea pigs are highly susceptible to the WE strain (LD_50_ < 1 plaque-forming unit [PFU]), while the mouse-adapted ARM strain failed to kill these animals at a dose >6 log_10_ PFU [6].

Previously, virus overlay protein-binding assays (VOPRAs) have demonstrated that pathogenic OW arenaviruses LCMV-WE and LASV bound to its major cellular receptor, α-DG, with higher affinity and efficiency than apathogenic LCMV-ARM and Mobala virus, MOBV, a non-pathogenic genetic relative of LASV [5,73,74]. However, at least for LCMV, the attachment efficacy of infectious particles of both strains, WE and ARM, was similar in polarized epithelial Caco-2 cells [60] and both LCMV strains replicated efficiently in BHK-21 cells [75]. Likewise, WE and ARM had similar replication kinetics in human and murine monocytes/macrophages. However, only LCMV-ARM and MOPV induced strong TLR-2/Mal-dependent cytokine responses in these cells while LCMV-WE, immunosuppressive LCMV Clone 13, and LASV did not [56]. This suggests that binding to α-DG measured by a VOPRA and replication efficiency measured by infectious assays in different cell types do not correlate with the ability to induce innate immunity and pro-inflammatory cytokine responses. Recent findings indicate that additional attachment and/or entry factors, DC-SIGN, LSECtin, Axl, TIM-1, and Tyro3, can contribute to the virus entry in an α-DG-independent manner [40,41,42,43,44]

Although there are few amino acid differences in the glycoprotein of LCMV-ARM and LCMV-WE, the result is a striking difference in affinity binding to α-DG, the major cellular receptor for LCMV and LASV [5,26,76]. In DCs that heavily express α-DG, LCMV-WE binds at high affinity while LCMV-ARM binds at low affinity [76,77]. LCMV-WE entry is dependent on α-DG, induces high levels of viral antigen expression, infiltration of white pulp area in the spleen [78], and ablation of CTL response [79]. High viral load at an early stage of the infection affected the cyto/chemokine expression profile and disrupted RNA sensing pathways. Release of immunosuppressive molecules, and suppression of MHC class II and co-stimulatory factors, additionally contributed to poor T cell responses and establishment of a persistent infection [24,80,81]. In a recent study, Xu et al. found that high-affinity α-DG receptor binding increased virus entry but was associated with IFN-I resistance, triggering innate affinity escape and impaired T cell immunity [82]. In contrast, the LCMV-ARM negligibly bound the α-DG receptor, infected limited numbers of DCs, and generated robust LCMV-specific T cell responses in mice [78,80,81].

In hepatocytes, monocytes, macrophages, epithelial, and other cells that utilize cell surface binding receptors other than α-DG (e.g., heparin sulfate, DC-SIGN, LSECtin, Axl-1, TIM-1, and Tyro3), LCMV-WE and LCMV-ARM bind at a similar efficiency and have comparable replication kinetics. However, in these cells, LCMV-ARM and MOPV infections resulted in TLR2/Mal-dependent innate sensing, induction of NF-κB-mediated pro-inflammatory cytokines, robust virus-specific T cell responses, and effective virus control. In contrast, infection with pathogenic arenaviruses, LCMV-WE and LASV, was associated with poor innate sensing, loss of NF-κB signaling, impaired IFN, and the down-regulation of pro-inflammatory responses resulted in progressed disease [27,28,56,57,58,83]. Accumulated evidence suggests that while tissue-specific patterns of α-DG receptor binding affinity contribute to LCMV and LASV virus entry and virus load, innate sensing, NF-κB signaling, and induction of pro-inflammatory responses seem to be the main driving forces of effective adaptive immunity. In addition, Z proteins of LCMV and LASV can affect RIG-I-dependent sensing and host innate immunity in a strain-specific manner [84].

Initial reports provided conflicting results on membrane cholesterol involvement in LCMV entry [34,85]. In our experiments, depletion of plasma membrane cholesterol and disruption of lipid rafts with Mβ-CD reduced replication of both strains, WE and ARM, in line with previously published results [62,85]. However, we observed greater sensitivity of LCMV-WE to Mβ-CD treatment compared with LCMV-ARM (Figure 1). Interestingly, α-DG is not associated with lipid rafts. Unsurprisingly, we were also unable to detect α-DG associated with lipid rafts but are unable to rule out technical difficulties in α-DG staining of Vero cells, but these cells do express high levels of Axl and TIM-1 on their cell surface [40]. Previously, Rojek JM et al. used a similar protocol for cholesterol depletion and found that upon cholesterol replenishment, there is a reversal of the inhibitory effect of Mβ-CD on LCMV infection, indicating that cell membrane cholesterol depletion was likely responsible for the virus replication inhibition [21]. Interestingly, while LCMV binding to cellular α-DG was cholesterol-independent, occurring in non-raft membranes, LCMV internalization was dependent on membrane cholesterol [62]. The impact of Mβ-CD treatment on virus particles cannot be excluded and is not addressed in this study. Further investigation may shed light on the effects of cholesterol depletion on unconventional receptor usage by LCMV because the antiviral mechanism of cholesterol depletion on LCMV entry is not clearly understood.

After cell entry, LCMV is sorted into vesicles and traffics through the endocytic machinery to deliver the virus cargo to late endosomes for fusion and the release of viral ribonuclear proteins (an association of viral proteins and viral RNA) into the cytoplasm prior to the formation of viral RTCs. Evidence from two relevant human cell lines demonstrates clear differences in NF-κB and IRF activation patterns between LCMV-ARM and LCMV-WE (Figure 2). The fold changes are smaller in THP1 cells, due to poor replication in non-differentiated monocytes, and experiments in matured macrophages confirmed strong NF-κB activation in ARM-infected cells vs WE-infected cells [56].

Accumulated evidence demonstrates a role for TLR-2 in sensing mammalian arenaviruses with different pathogenic potential [56,86,87,88,89,90]. In RNA silencing experiments, we confirmed that TLR-2 is required for IL-6 expression in ARM-infected cells (Figure 2). A viral glycoprotein was identified as a TLR-2 ligand for the New World Arenavirus Junín (JUNV) [87]. Viral glycoproteins of the OW arenaviruses should also be considered as TLR-2 ligands. However, in contrast to JUNV, TLR2/CD14/Mal-dependent signaling induced by LCMV-ARM and MOPV required virus internalization and replication [56], indicating a more complex interaction with TLR-2 for the OW arenaviruses.

Anti-TLR-2 antibody blocked the ability of non-pathogenic OW arenaviruses to induce pro-inflammatory cytokines and chemokines [56]. However, antibody treatment did not affect virus internalization and replication. This suggests that virus interaction with TLR-2 is essential for triggering cytokine signaling but is not required for virus entry and fusion. Virus interaction with the cell surface is a complex multi-step process [91]. After attachment to the cell surface, lateral movement along the plasma membrane results in receptor clustering (as observed for LCMV-ARM with TLR-2 and CD14 [56]), activation of cellular signaling pathways, and the initiation of endocytosis. Notably, inactivation of LCMV-ARM by UV or heat treatment abolished TLR2-dependent cytokine production [56]. This indicates that in addition to triggering TLR-2-dependent cytokine signaling on the cell surface, interactions between LCMV and TLR-2 in early endosomes contribute to the induction of pro-inflammatory cytokines in LCMV-ARM- but not LCMV-WE-infected cells. Mechanisms of this interaction warrant further investigation.

In α-DG-deficient cells (THP-1, HEK293T), LCMV-WE and LCMV-ARM replicate with similar kinetics. However, LCMV-ARM replication in these cells was associated with the induction of NF-κB-mediated pro-inflammatory cytokine responses, while LCMV-WE was not [56]. The phenotype of LCMV-WE compared to LCMV-ARM infection resembles LASV and MOPV infection, respectively, in validated animal models [6]. It has also been observed that there is a much greater inhibitory effect following ammonium chloride on MOPV compared to LASV replication in cell cultures [32]. Similarly, in the current study, bafilomycin A1 inhibition was significantly more impactful to LCMV-ARM infection, indicating greater sensitivity of LCMV-ARM to pH increase in endosomal compartments compared to LCMV-WE infection (Figure 1G). This study did not measure fusion kinetics. We suggest that in contrast to LASV and LCMV-WE infections, which may fuse in late endosomes/lysosomes, LCMV-ARM can fuse in a higher pH environment, in “earlier” endosomes, providing an advantage in viral release and contributing to innate immune sensing. Confocal staining with the late endosomal marker, Rab7, provided evidence for prolonged co-staining in LCMV-WE-infected cells compared to LCMV-ARM-infected cells (Figure 5).

In the case of LASV infection, intracellular receptor switching and binding to LAMP-1 [39] resulted in fusion at a less acidic pH [68]. While LCMV infection does not require LAMP-1, an intracellular switch to binding the lysosomal CD164 facilitated LCMV-ARM fusion at a low pH [92]. At present, there is no evidence for CD164 switch in LCMV-WE54 infection.

A low pH of endosomes/lysosomes is a major force triggering fusion events and the release of viral RNPs into the cytosol. Upon release, foreign RNA molecules are sensed by TLR-7/TLR-9 and by a cytosolic RIG-I. The RNA-sensing TLRs are located in a strategic position, within endosomes/lysosomes, to encounter foreign RNA molecules released from viral particles. Interestingly, a low pH environment is also necessary for the proper function of these receptors since acidification-inhibiting drugs (e.g., bafilomycin A1 or NH_4_Cl) reduced the activation of TLR7/TLR9 [93,94]. We previously documented a greater sensitivity of MOPV infection to NH_4_Cl treatment compared to LASV infection in Vero cells [32]. Similarly, LCMV-ARM infection in Vero E6 cells was more sensitive to bafilomycin treatment than infection with LCMV-WE (Figure 1). Different sensitivities of pathogenic versus non-pathogenic OW arenaviruses to pH increase in the late endosomes/lysosomes can affect the viral RNP release kinetics, RTC formation, and activation patterns of endosomal TLR-7/TLR-9 and cytosolic RIG-I. These factors are also crucially involved in the activation of antigen-presenting cells and T cell stimulation.

The enhanced innate immune activation caused by LCMV-ARM has applicability to cancer virotherapy. Non-oncolytic LCMV with preferential cancer tropism and antibody escape mechanisms is a promising tool to induce tumor-specific activation within the tumor microenvironment [95]. Restricted replication of LCMV in tumor tissues induced immune surveillance and IFN-I-dependent tumor regression [96]. In an in vivo human melanoma model, LCMV treatment up-regulated the chemokine ligand 5 (CCL5, RANTES), supported T cell functionality, promoted NK cell infiltration, and led to immune-mediated melanoma regression [97].

By electron microscopy, LCMV was detected in early and late endosomal compartments within 10–20 min after internalization, resembling influenza A virus entry kinetics [34,98]. In our confocal experiments, at a high MOI, 30 min after infection, about 10–20% cells stained positively for LCMV NP. A major limitation of confocal microscopy to observe early entry events with the available anti-NP-labeled antibody seems to be the dependance of strong signals on the formation of RTCs, an event that follows fusion. As a result, minimal co-staining was observed between viral NP signal and endosomal markers (Figure 4, Figure 5 and Figure 6). Still, when infected cells were co-stained with markers of early endosomes (EAA1 and Rab5) and TLR-2 to home in this compartment, EEA1 and TLR-2 co-localization seemed to increase in ARM- but not WE-infected cells (Figure 4). In line with previous observations by Quirin et al. [34], LCMV-WE had limited evidence of interaction with early endosomes and TLR-2.

Using single-cell vitalization tools, small GTPase Rab5 was co-localized not only with NP but also with GPC, matrix Z protein, and S RNA at the peak of virus production [99,100]. The authors proposed that Rab5c is required for efficient virus production at the late step of the infection coordinating trafficking and/or assembly of viral components to the budding sites on cell membranes [100]. A previous study using a constitutively inactivate (DN) mutant version of Rab5 also documented involvement of Rab5 in the replication of recombinant LCMV expressing LASV GPC (rLCMV-LASVGP) [35]. However, replication of LCMV-WE was not affected in cells transfected with DN Rab5 and DN Arf6 [34]. In the active form, the small GTPase Arf6 is involved in clathrin-coated pit formation and a variety of endocytic functions related to vesicle trafficking and early endosomes [101]. Based on our results (Figure 3) and previous observations, there is a growing body of evidence that LCMV-WE intracellular trafficking avoids Rab5/EEA1-positive early endosomes and the virus escapes recognition by PRRs associated with this sub-cellular compartment (e.g., TLR-2).

Both strains of LCMV had similar co-staining patterns with markers of late endosomes/lysosomes, although there was evidence of earlier transient co-staining for ARM-infected cells and prolonged co-staining at a later stage of infection for WE-infected cells (Figure 4 and Figure 5). Infection of cells with LCMV-WE and rLCMV-LASVGP was not affected by DN Rab7 [34,35]. Similarly, LCMV replication was not affected in LAMP-1-deficient cells [39]. It seems that functional Rab7 and LAMP-1 are not required for the replication of LCMV.

To elicit MyD88/Mal-dependent pro-inflammatory responses, IRAK-4 is recruited to phosphorylate IRAK-1 and IRAK-2, to further activate the down-stream cascade via the E3 ubiquitin ligase TRAF-6 and TAK-1 phosphorylation of IKKβ, resulting in IκB degradation and release of NF-κB [102]. LCMV-WE infection of murine macrophages did not result in co-localization of viral NP with IRAK-1 (Figure 6A). Interestingly, there was increased degradation of IRAK-1 in LCMV-ARM- but not WE-infected cells (Figure 6B). Taking into consideration the suppression of NF-κB in LCMV-WE-infected cells [56], this co-staining pattern seems to suggest this interaction is a mediator of the immune activation phenotype of LCMV-ARM.

In summary, this study demonstrated differences in terms of intracellular trafficking and sensitivity to pharmacological entry/fusion inhibition between ARM and WE, two strains of LCMV with different pathogenic potential in NHPs. These differences seem to play a role in the different innate sensing of LCMV-ARM and -WE. LCMV-WE is more susceptible to cholesterol depletion than LCMV-ARM. Conversely, LCMV-Arm is more reliant on endosomal acidification and induces more robust NF-κB and IRF responses. We hypothesize that this may be due to an increased interaction of LCMV-ARM and TLR7/TLR9 in the late endosomes, leading to more TLR- and RIG-I-based immune activation in contrast to pathogenic LCMV-WE. The mechanistic details of this trafficking pathway warrant further investigation.

## Data Availability

The original contributions presented in the study are included in the article, and further inquiries can be directed to the corresponding author.

## References

[1] Bonthius D.J. (2012). Lymphocytic Choriomeningitis Virus: An Underrecognized Cause of Neurologic Disease in the Fetus, Child, and Adult. Seminars in Pediatric Neurology.

[2] Lapošová K., Pastoreková S., Tomášková J. (2013). Lymphocytic Choriomeningitis Virus: Invisible but Not Innocent. Acta Virol..

[3] Radoshitzky S.R., Buchmeier M.J., Charrel R.N., Gonzalez J.-P.J., Günther S., Hepojoki J., Kuhn J.H., Lukashevich I.S., Romanowski V., Salvato M.S. (2023). ICTV Virus Taxonomy Profile: Arenaviridae 2023. J. Gen. Virol..

[4] Price M.E., Fisher-Hoch S.P., Craven R.B., McCormick J.B. (1988). A Prospective Study of Maternal and Fetal Outcome in Acute Lassa Fever Infection during Pregnancy. Br. Med. J..

[5] Cao W., Henry M.D., Borrow P., Yamada H., Elder J.H., Ravkov E.V., Nichol S.T., Compans R.W., Campbell K.P., Oldstone M.B. (1998). Identification of α-Dystroglycan as a Receptor for Lymphocytic Choriomeningitis Virus and Lassa Fever Virus. Science.

[6] Peters C.J., Jahrling P.B., Liu C.T., Kenyon R.H., McKee K.T., Barrera Oro J.G. (1987). Experimental Studies of Arenaviral Hemorrhagic Fevers. Arenaviruses: Biology and Immunotherapy.

[7] Zapata J.C., Pauza C.D., Djavani M.M., Rodas J.D., Moshkoff D., Bryant J., Ateh E., Garcia C., Lukashevich I.S., Salvato M.S. (2011). Lymphocytic Choriomeningitis Virus (LCMV) Infection of Macaques: A Model for Lassa Fever. Antivir. Res..

[8] Albariño C.G., Palacios G., Khristova M.L., Erickson B.R., Carroll S.A., Comer J.A., Hui J., Briese T., George K.S., Ksiazek T.G. (2010). High Diversity and Ancient Common Ancestry of Lymphocytic Choriomeningitis Virus. Emerg. Infect. Dis..

[9] Arruda L.B., Haider N., Olayemi A., Simons D., Ehichioya D., Yinka-Ogunleye A., Ansumana R., Thomason M.J., Asogun D., Ihekweazu C. (2021). The Niche of One Health Approaches in Lassa Fever Surveillance and Control. Ann. Clin. Microbiol. Antimicrob..

[10] Bowen M.D., Rollin P.E., Ksiazek T.G., Hustad H.L., Bausch D.G., Demby A.H., Bajani M.D., Peters C.J., Nichol S.T. (2000). Genetic Diversity among Lassa Virus Strains. J. Virol..

[11] Kouadio L., Nowak K., Akoua-Koffi C., Weiss S., Allali B.K., Witkowski P.T., Krüger D.H., Couacy-Hymann E., Calvignac-Spencer S., Leendertz F.H. (2015). Lassa Virus in Multimammate Rats, Côte d’Ivoire, 2013. Emerg. Infect. Dis..

[12] Manning J.T., Forrester N., Paessler S. (2015). Lassa Virus Isolates from Mali and the Ivory Coast Represent an Emerging Fifth Lineage. Front. Microbiol..

[13] Safronetz D., Lopez J.E., Sogoba N., Traore S.F., Raffel S.J., Fischer E.R., Ebihara H., Branco L., Garry R.F., Schwan T.G. (2010). Detection of Lassa Virus, Mali. Emerg. Infect. Dis..

[14] Whitmer S.L., Strecker T., Cadar D., Dienes H.-P., Faber K., Patel K., Brown S.M., Davis W.G., Klena J.D., Rollin P.E. (2018). New Lineage of Lassa Virus, Togo, 2016. Emerg. Infect. Dis..

[15] McCormick J.B., Webb P.A., Krebs J.W., Johnson K.M., Smith E.S. (1987). A Prospective Study of the Epidemiology and Ecology of Lassa Fever. J. Infect. Dis..

[16] Garry R.F. (2023). Lassa Fever—The Road Ahead. Nat. Rev. Microbiol..

[17] Fischer S.A., Graham M.B., Kuehnert M.J., Kotton C.N., Srinivasan A., Marty F.M., Comer J.A., Guarner J., Paddock C.D., DeMeo D.L. (2006). Transmission of Lymphocytic Choriomeningitis Virus by Organ Transplantation. N. Engl. J. Med..

[18] MacNeil A., Ströher U., Farnon E., Campbell S., Cannon D., Paddock C.D., Drew C.P., Kuehnert M., Knust B., Gruenenfelder R. (2012). Solid Organ Transplant–Associated Lymphocytic Choriomeningitis, United States, 2011. Emerg. Infect. Dis..

[19] Zinkernagel R.M., Haenseler E., Leist T., Cerny A., Hengartner H., Althage A. (1986). T Cell-Mediated Hepatitis in Mice Infected with Lymphocytic Choriomeningitis Virus. Liver Cell Destruction by H-2 Class I-Restricted Virus-Specific Cytotoxic T Cells as a Physiological Correlate of the 51Cr-Release Assay?. J. Exp. Med..

[20] Lang P.A., Recher M., Honke N., Scheu S., Borkens S., Gailus N., Krings C., Meryk A., Kulawik A., Cervantes-Barragan L. (2010). Tissue Macrophages Suppress Viral Replication and Prevent Severe Immunopathology in an interferon-I-dependent Manner in Mice. Hepatology.

[21] Beier J.I., Jokinen J.D., Holz G.E., Whang P.S., Martin A.M., Warner N.L., Arteel G.E., Lukashevich I.S. (2015). Novel Mechanism of Arenavirus-Induced Liver Pathology. PLoS ONE.

[22] Oldstone M.B. (1996). Principles of Viral Pathogenesis. Cell.

[23] Matullo C.M., O’Regan K.J., Hensley H., Curtis M., Rall G.F. (2010). Lymphocytic Choriomeningitis Virus-Induced Mortality in Mice Is Triggered by Edema and Brain Herniation. J. Virol..

[24] Ware B.C., Sullivan B.M., LaVergne S., Marro B.S., Egashira T., Campbell K.P., Elder J., Oldstone M.B. (2019). A Unique Variant of Lymphocytic Choriomeningitis Virus That Induces Pheromone Binding Protein MUP: Critical Role for CTL. Proc. Natl. Acad. Sci. USA.

[25] Sevilla N., Domingo E., de la Torre J.C. (2002). Contribution of LCMV towards Deciphering Biology of Quasispecies in Vivo. Arenaviruses II Mol. Pathog. Arenavirus Infect..

[26] Sevilla N., Kunz S., Holz A., Lewicki H., Homann D., Yamada H., Campbell K.P., de la Torre J.C., Oldstone M.B. (2000). Immunosuppression and Resultant Viral Persistence by Specific Viral Targeting of Dendritic Cells. J. Exp. Med..

[27] Lukashevich I.S., Tikhonov I., Rodas J.D., Zapata J.C., Yang Y., Djavani M., Salvato M.S. (2003). Arenavirus-Mediated Liver Pathology: Acute Lymphocytic Choriomeningitis Virus Infection of Rhesus Macaques Is Characterized by High-Level Interleukin-6 Expression and Hepatocyte Proliferation. J. Virol..

[28] Lukashevich I.S., Rodas J.D., Tikhonov I.I., Zapata J.C., Yang Y., Djavani M., Salvato M.S. (2004). LCMV-Mediated Hepatitis in Rhesus Macaques: WE but Not ARM Strain Activates Hepatocytes and Induces Liver Regeneration. Arch. Virol..

[29] Rodas J.D., Lukashevich I.S., Zapata J.C., Cairo C., Tikhonov I., Djavani M., Pauza C.D., Salvato M.S. (2004). Mucosal Arenavirus Infection of Primates Can Protect Them from Lethal Hemorrhagic Fever. J. Med. Virol..

[30] Stein D.R., Warner B.M., Audet J., Soule G., Siragam V., Sroga P., Griffin B.D., Leung A., Grolla A., Tierney K. (2021). Differential Pathogenesis of Closely Related 2018 Nigerian Outbreak Clade III Lassa Virus Isolates. PLoS Pathog..

[31] Yun N.E., Ronca S., Tamura A., Koma T., Seregin A.V., Dineley K.T., Miller M., Cook R., Shimizu N., Walker A.G. (2016). Animal Model of Sensorineural Hearing Loss Associated with Lassa Virus Infection. J. Virol..

[32] Glushakova S.E., Lukashevich I.S. (1989). Early Events in Arenavirus Replication Are Sensitive to Lysosomotropic Compounds. Arch. Virol..

[33] Pasqual G., Rojek J.M., Masin M., Chatton J.-Y., Kunz S. (2011). Old World Arenaviruses Enter the Host Cell via the Multivesicular Body and Depend on the Endosomal Sorting Complex Required for Transport. PLoS Pathog..

[34] Quirin K., Eschli B., Scheu I., Poort L., Kartenbeck J., Helenius A. (2008). Lymphocytic Choriomeningitis Virus Uses a Novel Endocytic Pathway for Infectious Entry via Late Endosomes. Virology.

[35] Rojek J.M., Sanchez A.B., Nguyen N.T., de la Torre J.-C., Kunz S. (2008). Different Mechanisms of Cell Entry by Human-Pathogenic Old World and New World Arenaviruses. J. Virol..

[36] Iwasaki M., Ngo N., de la Torre J.C. (2014). Sodium Hydrogen Exchangers Contribute to Arenavirus Cell Entry. J. Virol..

[37] Oppliger J., Torriani G., Herrador A., Kunz S. (2016). Lassa Virus Cell Entry via Dystroglycan Involves an Unusual Pathway of Macropinocytosis. J. Virol..

[38] Cohen-Dvashi H., Cohen N., Israeli H., Diskin R. (2015). Molecular Mechanism for LAMP1 Recognition by Lassa Virus. J. Virol..

[39] Jae L.T., Raaben M., Herbert A.S., Kuehne A.I., Wirchnianski A.S., Soh T.K., Stubbs S.H., Janssen H., Damme M., Saftig P. (2014). Lassa Virus Entry Requires a Trigger-Induced Receptor Switch. Science.

[40] Brouillette R.B., Phillips E.K., Patel R., Mahauad-Fernandez W., Moller-Tank S., Rogers K.J., Dillard J.A., Cooney A.L., Martinez-Sobrido L., Okeoma C. (2018). TIM-1 Mediates Dystroglycan-Independent Entry of Lassa Virus. J. Virol..

[41] Fedeli C., Torriani G., Galan-Navarro C., Moraz M.-L., Moreno H., Gerold G., Kunz S. (2018). Axl Can Serve as Entry Factor for Lassa Virus Depending on the Functional Glycosylation of Dystroglycan. J. Virol..

[42] Goncalves A.-R., Moraz M.-L., Pasquato A., Helenius A., Lozach P.-Y., Kunz S. (2013). Role of DC-SIGN in Lassa Virus Entry into Human Dendritic Cells. J. Virol..

[43] Shimojima M., Kawaoka Y. (2012). Cell Surface Molecules Involved in Infection Mediated by Lymphocytic Choriomeningitis Virus Glycoprotein. J. Vet. Med. Sci..

[44] Shimojima M., Ströher U., Ebihara H., Feldmann H., Kawaoka Y. (2012). Identification of Cell Surface Molecules Involved in Dystroglycan-Independent Lassa Virus Cell Entry. J. Virol..

[45] Fedeli C., Moreno H., Kunz S. (2020). The Role of Receptor Tyrosine Kinases in Lassa Virus Cell Entry. Viruses.

[46] Lukashevich I.S., Paessler S., de la Torre J.C. (2019). Lassa Virus Diversity and Feasibility for Universal Prophylactic Vaccine. F1000Research.

[47] Amara A., Mercer J. (2015). Viral Apoptotic Mimicry. Nat. Rev. Microbiol..

[48] Bhattacharyya S., Zagórska A., Lew E.D., Shrestha B., Rothlin C.V., Naughton J., Diamond M.S., Lemke G., Young J.A. (2013). Enveloped Viruses Disable Innate Immune Responses in Dendritic Cells by Direct Activation of TAM Receptors. Cell Host Microbe.

[49] Moller-Tank S., Maury W. (2014). Phosphatidylserine Receptors: Enhancers of Enveloped Virus Entry and Infection. Virology.

[50] Fan L., Briese T., Lipkin W.I. (2010). Z Proteins of New World Arenaviruses Bind RIG-I and Interfere with Type I Interferon Induction. J. Virol..

[51] Jiang X., Huang Q., Wang W., Dong H., Ly H., Liang Y., Dong C. (2013). Structures of Arenaviral Nucleoproteins with Triphosphate dsRNA Reveal a Unique Mechanism of Immune Suppression. J. Biol. Chem..

[52] Pythoud C., Rodrigo W.S.I., Pasqual G., Rothenberger S., Martínez-Sobrido L., de la Torre J.C., Kunz S. (2012). Arenavirus Nucleoprotein Targets Interferon Regulatory Factor-Activating Kinase IKKε. J. Virol..

[53] Xing J., Ly H., Liang Y. (2015). The Z Proteins of Pathogenic but Not Nonpathogenic Arenaviruses Inhibit RIG-I-like Receptor-Dependent Interferon Production. J. Virol..

[54] Borrow P., Martínez-Sobrido L., De la Torre J.C. (2010). Inhibition of the Type I Interferon Antiviral Response during Arenavirus Infection. Viruses.

[55] Trinchieri G. (2012). Lymphocyte Choriomeningitis Virus Plays Hide-and-Seek with Type 1 Interferon. Cell Host Microbe.

[56] Hayes M.W., Carrion R., Nunneley J., Medvedev A.E., Salvato M.S., Lukashevich I.S. (2012). Pathogenic Old World Arenaviruses Inhibit TLR2/Mal-Dependent Proinflammatory Cytokines in Vitro. J. Virol..

[57] Carrion R., Brasky K., Mansfield K., Johnson C., Gonzales M., Ticer A., Lukashevich I., Tardif S., Patterson J. (2007). Lassa Virus Infection in Experimentally Infected Marmosets: Liver Pathology and Immunophenotypic Alterations in Target Tissues. J. Virol..

[58] Lukashevich I.S., Maryankova R., Vladyko A.S., Nashkevich N., Koleda S., Djavani M., Horejsh D., Voitenok N.N., Salvato M.S. (1999). Lassa and Mopeia Virus Replication in Human Monocytes/Macrophages and in Endothelial Cells: Different Effects on IL-8 and TNF-α Gene Expression. J. Med. Virol..

[59] Lukashevich I.S., Carrion R., Salvato M.S., Mansfield K., Brasky K., Zapata J., Cairo C., Goicochea M., Hoosien G.E., Ticer A. (2008). Safety, Immunogenicity, and Efficacy of the ML29 Reassortant Vaccine for Lassa Fever in Small Non-Human Primates. Vaccine.

[60] Warner N.L., Jokinen J.D., Beier J.I., Sokoloski K.J., Lukashevich I.S. (2018). Mammarenaviral Infection Is Dependent on Directional Exposure to and Release from Polarized Intestinal Epithelia. Viruses.

[61] Cosset F.-L., Marianneau P., Verney G., Gallais F., Tordo N., Pécheur E.-I., ter Meulen J., Deubel V., Bartosch B. (2009). Characterization of Lassa Virus Cell Entry and Neutralization with Lassa Virus Pseudoparticles. J. Virol..

[62] Rojek J.M., Perez M., Kunz S. (2008). Cellular Entry of Lymphocytic Choriomeningitis Virus. J. Virol..

[63] Danthi P., Chow M. (2004). Cholesterol Removal by Methyl-β-Cyclodextrin Inhibits Poliovirus Entry. J. Virol..

[64] Lambert D., O’NEILL C.A., Padfield P.J. (2005). Depletion of Caco-2 Cell Cholesterol Disrupts Barrier Function by Altering the Detergent Solubility and Distribution of Specific Tight-Junction Proteins. Biochem. J..

[65] Wang M., Jokinen J., Tretyakova I., Pushko P., Lukashevich I.S. (2018). Alphavirus Vector-Based Replicon Particles Expressing Multivalent Cross-Protective Lassa Virus Glycoproteins. Vaccine.

[66] Idrees S., Chen H., Panth N., Paudel K.R., Hansbro P.M. (2024). Exploring Viral–Host Protein Interactions as Antiviral Therapies: A Computational Perspective. Microorganisms.

[67] Iwasaki M., Minder P., Cai Y., Kuhn J.H., Yates III J.R., Torbett B.E., de la Torre J.C. (2018). Interactome Analysis of the Lymphocytic Choriomeningitis Virus Nucleoprotein in Infected Cells Reveals ATPase Na^+^/K^+^ Transporting Subunit Alpha 1 and Prohibitin as Host-Cell Factors Involved in the Life Cycle of Mammarenaviruses. PLoS Pathog..

[68] Hulseberg C.E., Fénéant L., Szymańska K.M., White J.M. (2018). Lamp1 Increases the Efficiency of Lassa Virus Infection by Promoting Fusion in Less Acidic Endosomal Compartments. MBio.

[69] Spearman P. (2018). Viral Interactions with Host Cell Rab GTPases. Small GTPases.

[70] Brown J., Wang H., Hajishengallis G.N., Martin M. (2011). TLR-Signaling Networks: An Integration of Adaptor Molecules, Kinases, and Cross-Talk. J. Dent. Res..

[71] Kawagoe T., Sato S., Matsushita K., Kato H., Matsui K., Kumagai Y., Saitoh T., Kawai T., Takeuchi O., Akira S. (2008). Sequential Control of Toll-like Receptor–Dependent Responses by IRAK1 and IRAK2. Nat. Immunol..

[72] McCormick J.B., Walker D.H., King I.J., Webb P.A., Elliott L.H., Whitfield S.G., Johnson K.M. (1986). Lassa Virus Hepatitis: A Study of Fatal Lassa Fever in Humans. Am. J. Trop. Med. Hyg..

[73] Kunz S., Rojek J.M., Kanagawa M., Spiropoulou C.F., Barresi R., Campbell K.P., Oldstone M.B. (2005). Posttranslational Modification of α-Dystroglycan, the Cellular Receptor for Arenaviruses, by the Glycosyltransferase LARGE Is Critical for Virus Binding. J. Virol..

[74] Kunz S., Rojek J.M., Perez M., Spiropoulou C.F., Oldstone M.B. (2005). Characterization of the Interaction of Lassa Fever Virus with Its Cellular Receptor α-Dystroglycan. J. Virol..

[75] Hastie K.M., Igonet S., Sullivan B.M., Legrand P., Zandonatti M.A., Robinson J.E., Garry R.F., Rey F.A., Oldstone M.B., Saphire E.O. (2016). Crystal Structure of the Prefusion Surface Glycoprotein of the Prototypic Arenavirus LCMV. Nat. Struct. Mol. Biol..

[76] Kunz S., Sevilla N., McGavern D.B., Campbell K.P., Oldstone M.B. (2001). Molecular Analysis of the Interaction of LCMV with Its Cellular Receptor α-Dystroglycan. J. Cell Biol..

[77] Sevilla N., Kunz S., McGavern D., Oldstone M.B.A. (2003). Infection of Dendritic Cells by Lymphocytic Choriomeningitis Virus. Dendritic Cells Virus Infect..

[78] Smelt S.C., Borrow P., Kunz S., Cao W., Tishon A., Lewicki H., Campbell K.P., Oldstone M.B. (2001). Differences in Affinity of Binding of Lymphocytic Choriomeningitis Virus Strains to the Cellular Receptor α-Dystroglycan Correlate with Viral Tropism and Disease Kinetics. J. Virol..

[79] Ng C.T., Oldstone M.B. (2012). Infected CD8α− Dendritic Cells Are the Predominant Source of IL-10 during Establishment of Persistent Viral Infection. Proc. Natl. Acad. Sci. USA.

[80] Borrow P., Evans C.F., Oldstone M.B. (1995). Virus-Induced Immunosuppression: Immune System-Mediated Destruction of Virus-Infected Dendritic Cells Results in Generalized Immune Suppression. J. Virol..

[81] Sullivan B.M., Emonet S.F., Welch M.J., Lee A.M., Campbell K.P., de la Torre J.C., Oldstone M.B. (2011). Point Mutation in the Glycoprotein of Lymphocytic Choriomeningitis Virus Is Necessary for Receptor Binding, Dendritic Cell Infection, and Long-Term Persistence. Proc. Natl. Acad. Sci. USA.

[82] Xu H.C., Pandey P., Ward H., Gorzkiewicz M., Abromavičiūtė D., Tinz C., Müller L., Meyer C., Pandyra A.A., Yavas A. (2024). High-Affinity–Mediated Viral Entry Triggers Innate Affinity Escape Resulting in Type I IFN Resistance and Impaired T Cell Immunity. J. Immunol..

[83] Namineni S., O’Connor T., Faure-Dupuy S., Johansen P., Riedl T., Liu K., Xu H., Singh I., Shinde P., Li F. (2020). A Dual Role for Hepatocyte-Intrinsic Canonical NF-κB Signaling in Virus Control. J. Hepatol..

[84] Huang Q., Liu X., Brisse M., Ly H., Liang Y. (2020). Effect of Strain Variations on Lassa Virus Z Protein-Mediated Human RIG-I Inhibition. Viruses.

[85] Shah W.A., Peng H., Carbonetto S. (2006). Role of Non-Raft Cholesterol in Lymphocytic Choriomeningitis Virus Infection via α-Dystroglycan. J. Gen. Virol..

[86] Cuevas C.D., Ross S.R. (2014). Toll-like Receptor 2-Mediated Innate Immune Responses against Junín Virus in Mice Lead to Antiviral Adaptive Immune Responses during Systemic Infection and Do Not Affect Viral Replication in the Brain. J. Virol..

[87] Cuevas C.D., Lavanya M., Wang E., Ross S.R. (2011). Junin Virus Infects Mouse Cells and Induces Innate Immune Responses. J. Virol..

[88] Zhou S., Cerny A.M., Bowen G., Chan M., Knipe D.M., Kurt-Jones E.A., Finberg R.W. (2010). Discovery of a Novel TLR2 Signaling Inhibitor with Anti-Viral Activity. Antivir. Res..

[89] Zhou S., Halle A., Kurt-Jones E.A., Cerny A.M., Porpiglia E., Rogers M., Golenbock D.T., Finberg R.W. (2008). Lymphocytic Choriomeningitis Virus (LCMV) Infection of CNS Glial Cells Results in TLR2-MyD88/Mal-Dependent Inflammatory Responses. J. Neuroimmunol..

[90] Zhou S., Kurt-Jones E.A., Mandell L., Cerny A., Chan M., Golenbock D.T., Finberg R.W. (2005). MyD88 Is Critical for the Development of Innate and Adaptive Immunity during Acute Lymphocytic Choriomeningitis Virus Infection. Eur. J. Immunol..

[91] Helenius A. (2018). Virus Entry: Looking Back and Moving Forward. J. Mol. Biol..

[92] Bakkers M.J., Moon-Walker A., Herlo R., Brusic V., Stubbs S.H., Hastie K.M., Saphire E.O., Kirchhausen T.L., Whelan S.P. (2022). CD164 Is a Host Factor for Lymphocytic Choriomeningitis Virus Entry. Proc. Natl. Acad. Sci. USA.

[93] Lee B.L., Barton G.M. (2014). Trafficking of Endosomal Toll-like Receptors. Trends Cell Biol..

[94] Wu J., Chen Z.J. (2014). Innate Immune Sensing and Signaling of Cytosolic Nucleic Acids. Annu. Rev. Immunol..

[95] Stachura P., Stencel O., Lu Z., Borkhardt A., Pandyra A.A. (2023). Arenaviruses: Old Viruses Present New Solutions for Cancer Therapy. Front. Immunol..

[96] Kalkavan H., Sharma P., Kasper S., Helfrich I., Pandyra A.A., Gassa A., Virchow I., Flatz L., Brandenburg T., Namineni S. (2017). Spatiotemporally Restricted Arenavirus Replication Induces Immune Surveillance and Type I Interferon-Dependent Tumour Regression. Nat. Commun..

[97] Bhat H., Zaun G., Hamdan T.A., Lang J., Adomati T., Schmitz R., Friedrich S.-K., Bergerhausen M., Cham L.B., Li F. (2020). Arenavirus Induced CCL5 Expression Causes NK Cell-Mediated Melanoma Regression. Front. Immunol..

[98] Baharom F., Thomas O.S., Lepzien R., Mellman I., Chalouni C., Smed-Sörensen A. (2017). Visualization of Early Influenza A Virus Trafficking in Human Dendritic Cells Using STED Microscopy. PLoS ONE.

[99] King B.R., Samacoits A., Eisenhauer P.L., Ziegler C.M., Bruce E.A., Zenklusen D., Zimmer C., Mueller F., Botten J. (2018). Visualization of Arenavirus RNA Species in Individual Cells by Single-Molecule Fluorescence in Situ Hybridization Suggests a Model of Cyclical Infection and Clearance during Persistence. J. Virol..

[100] Ziegler C.M., Bruce E.A., Kelly J.A., King B.R., Botten J.W. (2018). The Use of Novel Epitope-Tagged Arenaviruses Reveals That Rab5c-Positive Endosomal Membranes Are Targeted by the LCMV Matrix Protein. J. Gen. Virol..

[101] Hongu T., Kanaho Y. (2014). Activation Machinery of the Small GTPase Arf6. Adv. Biol. Regul..

[102] Blasius A.L., Beutler B. (2010). Intracellular Toll-like Receptors. Immunity.

